# The Compositionally Distinct Cyanobacterial Biocrusts From Brazilian Savanna and Their Environmental Drivers of Community Diversity

**DOI:** 10.3389/fmicb.2019.02798

**Published:** 2019-12-17

**Authors:** Náthali Maria Machado-de-Lima, Vanessa Moreira Câmara Fernandes, Daniel Roush, Sergio Velasco Ayuso, Janaina Rigonato, Ferran Garcia-Pichel, Luis Henrique Zanini Branco

**Affiliations:** ^1^Microbiology Graduation Program, Department of Zoology and Botany, São Paulo State University (UNESP), São Paulo, Brazil; ^2^Center for Fundamental and Applied Microbiomics, Biodesign Institute, Arizona State University, Tempe, AZ, United States; ^3^Facultad de Agronomía, Instituto de Investigaciones Fisiológicas y Ecológicas Vinculadas a la Agricultura (IFEVA-CONICET), Universidad de Buenos Aires, Buenos Aires, Argentina; ^4^Center for Nuclear Energy in Agriculture (CENA), University of São Paulo (USP), Piracicaba, Brazil

**Keywords:** biocrust, *cerrado* savanna, cyanobacteria, *Leptolyngbya*, *Porphyrosiphon*, aridity

## Abstract

The last decade was marked by efforts to define and identify the main cyanobacterial players in biological crusts around the world. However, not much is known about biocrusts in Brazil’s tropical savanna (*cerrado*), despite the existence of environments favorable to their development and ecological relevance. We examined the community composition of cyanobacteria in biocrusts from six sites distributed in the Southeast of the country using high throughput sequencing of 16S rRNA and phylogenetic placement in the wider context of biocrusts from deserts. Sequences ascribable to 22 genera of cyanobacteria were identified. Although a significant proportion of sequences did not match those of known cyanobacteria, several clades of *Leptolyngbya* and *Porphyrosiphon* were found to be the most abundant. We identified significant differences in dominance and overall composition among the *cerrado* sites, much larger than within-site variability. The composition of *cerrado* cyanobacterial communities was distinct from those known in biocrusts from North American deserts. Among several environmental drivers considered, the opposing trend of annual precipitation and mean annual temperature best explained the variability in community composition within Brazilian biocrusts. Their compositional uniqueness speaks of the need for dedicated efforts to study the ecophysiology of tropical savanna biocrust and their roles in ecosystem function for management and preservation.

## Introduction

Biological soil crusts (BSCs or biocrusts) are microbial assemblages present at the top soil of several terrestrial ecosystems, especially in drylands. Biocrusts are typically composed of cyanobacteria (Garcia-Pichel et al., 2001) but sometimes also eukaryotic algae, lichens, or mosses (Bates et al., 2010b) as primary producers, accompanied by a variety of chemotrophic bacteria (da Rocha et al., 2015), archaea (Soule et al., 2009), and fungi (Bates et al., 2010a). Biocrusts are ecologically important biotic components of arid lands (see reviews by Eldridge and Greene, 1994; Belnap et al., 2016), but they can occur in a variety of climatic regions, colonizing places where sunlight reaches the soil surface or even temperate areas subject to disturbance (Gundlapally and Garcia-Pichel, 2006; Elbert et al., 2009; Pointing and Belnap, 2012). Cyanobacteria contribute to important soil functions within biocrusts, providing stability and protection against erosive forces (Belnap, 2003, 2005) and are also responsible for carbon and nitrogen fixation, enriching soils with macronutrients (Johnson et al., 2007) and micronutrients (Beraldi-Campesi et al., 2009). These contributions of cyanobacteria to the ecosystem highlight the importance of understanding their composition and function worldwide.

However, a majority of the studies on cyanobacteria have focused in arid or semiarid areas in North America (Garcia-Pichel et al., 2013; Couradeau et al., 2016; Fernandes et al., 2018), China (Zhang et al., 2016), Australia (Delgado-Baquerizo et al., 2018), Spain (Williams et al., 2016), and the Middle East (Abed et al., 2010; Hagemann et al., 2015; Nejidat et al., 2016). The bundle-forming morphogenus *Microcoleus* seems to be dominant worldwide, even in areas where studies only used microscopy or clone libraries, such as Chile (Baumann et al., 2018) and the hyperarid Atacama Desert (Patzelt et al., 2014). Arid land biocrusts are usually first colonized by *Microcoleus* species, which stabilize the soil and start fixing carbon (Garcia-Pichel and Wojciechowski, 2009), enriching the soil and allowing heterocytous nitrogen-fixing cyanobacteria, usually *Scytonema* sp. and *Tolypothrix* sp. to colonize. Other common cyanobacteria present in arid land crusts include species of *Nostoc*, *Calothrix*, *Chroococidiopsis*, *Leptolyngbya*, *Phormidium* and *Schizothrix*. Whenever compositional studies have been carried out in biocrust from other climates, apparently differentiated communities were encountered (Pushkareva et al., 2015; Muñoz-Martín et al., 2019).

In Brazil, cyanobacterial biocrusts are found in both arid and semiarid areas in the Northeast of the country, but also in the savanna biome called *cerrado*. This biome represents about 2 million km^2^ or 23% of the country’s land surface, surpassed only by the Amazonian forest (Ratter et al., 1997), but its natural areas (anthropized areas – urban and agricultural – and water bodies excluded) cover approximated 1.24 million km^2^ (Sano et al., 2007). Some of *cerrado* phytophysiognomies are more favorable to crust occurrence due to the absence of a developed canopy and these non-forest formations are estimated to cover some 0.8 million km^2^, corresponding to 68% of the total *cerrado* area, according to Sano et al. (2007). The *cerrado* climate is classified as semi-humid tropical and is one of the most humid savanna regions in the world but with a severe dry season during April-September. Average annual precipitation varies between 800 and 2,000 mm, and average annual temperatures between 18 and 28°C (Eiten, 1982). Preliminary analyses by microscopy revealed the possibility of finding species not previously reported for biocrusts. Considering the absence of prior biocrust work on the *cerrado*, we undertook a survey of six sites to study their cyanobacterial components. The results of such an approach allowed us to compare the biodiversity and composition of the cyanobacterial assemblages of crusts from different environments (savanna vs. desert). In addition, the influence of selected environmental factors on composition within *cerrado* biocrusts was also evaluated. Our study provides a necessary foundation for more detailed studies and a relevant source of information for management and restoration practices of disturbed areas (Giraldo-Silva et al., 2018), particularly in view of their compositional uniqueness, which makes extrapolation of knowledge obtained from desert biocrust uncertain.

## Materials and Methods

### Sampling

Biocrusts were sampled in six sites distributed in four preserved areas in the SE region of Brazil (Supplementary Figure S1): *Furnas do Bom Jesus* São Paulo State Park of (one site), *Vassununga* São Paulo State Park (one site), *Serra do Cipó* National Park (two sites: *Cipó* and *Capão*), and *Serra da Canastra* National Park (two sites: *Canastra* and *Zagaia*) (Table 1). Ten sequential equidistant samples were collected throughout a 200 m long transect with two parallel transects set at each site, encompassing a total of 120 samples for the study (10 samples/transect × two transects/site × six sites). Soil crusts were collected with a Petri dish (55 mm × 1 cm high) and transported to the laboratory where they were kept dry at −20°C until processing.

**Table 1:** Samples and sampling localities of biological crusts in *cerrado* savanna.

Sample code	Origin	GPS coordinates
FU1, FU2, FU3, FU4, FU5, FU6	*Furnas do Bom Jesus* State Park	20°14′S, 47°27′W
CI1, CI2, CI3, CI4, CI5, CI6	*Serra do Cipó* National Park – *Cipó*	19°20′S, 43°34′W
CP1, CP4, CP2, CP5, CP3, CP6.	*Serra do Cipó* National Park *– Capão*	19°20′S, 43°34′W
CA1, CA2, CA3, CA4, CA5, CA6	*Serra da Canastra* National Park – *Canastra*	20°21′S, 46°38′W
ZA1, ZA2, ZA3, ZA4, ZA5, ZA6	*Serra da Canastra* National Park *– Zagaia*	20°21′S, 46°38′W
VA1, VA2, VA3, VA4, VA5, VA6	*Vassununga* State Park	21°37′S, 47°37′W

### Library Preparation and Illumina Sequencing of the 16S rRNA Gene

Soil grains, rocks, and organic matter (plant roots and leaves) were manually removed, and 1 mg of the sampled biocrust was used for DNA extraction with MoBio Powersoil kit (Laboratories Inc., Carlsbad, CA, USA) according to the manufacturer’s instructions. The region V3-V4 of 16S rRNA gene (420 bp) was amplified with CYA 359 and 781a/b primers as described in Nübel et al. (1997), with an overhang Illumina adapter included in the primers. The PCR reaction contained 10 ng of eDNA, 0.2 μM of each primer, and 1x KAPA HiFi HotStart Ready Mix (KAPA Biosystems) to 25 μl of final volume. The PCR product from the samples 1–3, 4–6, and 7–10 of each transect were pooled and purified using AMPure XP purification kit (Beckman Coulter Inc., Brea, CA, USA). Afterwards, Illumina sequencing adapters and dual-index barcodes were added to the amplicon target using the Nextera XT Index Kit (Illumina, USA), according to the manufacturer’s instructions. The product was purified using AMPure XP purification kit, quantified with Qubit Fluorometric Quantitation (Thermofisher/Life Technology, USA). The samples were normalized and, then, pooled in an equimolar fashion. The preparation of the samples followed the Illumina guidelines for sequencing in a MiSeq platform (Illumina) available at Center of Nuclear Energy and Agriculture (ESALQ/USP) and using MiSeq Reagent kit v3. 2 × 300 cycle.

### Data Analysis Pipeline

The 16S rRNA gene forward and reverse sequences were paired using PANDAseq (Masella et al., 2012) and a fastq file was generated. The “QIIME 1.9” [Quantitative Insights into Microbial Ecology (Caporaso et al., 2010)] was used for further analyses. The script “multiple_split_libraries_fasq.py” was run without quality filtering, as quality filtering was done before pairing using Trimmomatic (Bolger et al., 2014). Next, the script “pick_open_reference_otus.py”, clustered reads into 97% self-similar operational taxonomic units (OTUs) using SUMAClust (Schloss, 2016) and SortMeRNA (Kopylova et al., 2012) in combination with the Greengenes 13_8 database (DeSantis et al., 2006). A filter was applied to the OTU table and only the OTUs that appeared in at least two samples were considered in the analyses. The OTU table also was used to do a rarefaction curve using “Chao1” and “goods_coverage” methods, through the script “alpha_diversity.py”, also in “QIIME 1.9”. All sequence datasets are publicly available through NCBI under the project “Diversity and ecology of cyanobacteria of biological soil crusts in Brazilian Savannah” (NCBI identification number: SRP137259).

### Operational Taxonomic Unit Taxonomic Assignment

OTUs were identified by comparison with taxonomic information provided by public databanks. OTUs that presented more than 199 reads were first identified using QIIME (based on SortMeRNA and the Green Genes 13_8 database – DeSantis et al., 2006) and then compared one by one with GenBank data (NCBI nr/nt) using the tool “Basic Local Alignment Search Tool” (Baumann et al., 1990) to refine the initial identification. Cyanobacterial OTUs’ taxonomic assignment at the genus and species level was further informed through phylogenetic placement in the cyanobacterial reference database, Cydrasil (v. rc1, https://github.com/FGPLab/cydrasil). The Cydrasil rc1 database contains 1,161 curated cyanobacterial 16S rRNA gene sequences that are at least 1,100 bp long and includes a phylogenetic tree generated using RAxML 8 (Berger and Stamatakis, 2011). Query cyanobacterial sequences were aligned to the reference alignment with PaPaRa (Berger and Stamatakis, 2011), placed into the reference tree using the RaxML8 Evolutionary Placement Algorithm (Berger and Stamatakis, 2011) without changing the tree topology, and visualized on the iTOL 3 server (Letunic and Bork, 2016). This procedure allowed to relate an OTU sequence to a well-curated specific phylogenetic group or clade in a database that is enriched in cyanobacterial sequences from biocrusts, and thus confirm (or correct) the initial taxonomic identification. In general, OTUs that were 95% similar to an identified sequence (following Yarza et al., 2014) and placed in a highly supported and well-defined clade composed of coherently identified sequences were considered pertaining to the same genus. OTUs that presented less than 94% of similarity to the closest sequence were not identified. OTUs related to a single genus name but distributed in different clades (as the cases of the polyphyletic genera *Leptolyngbya* and *Microcoleus*) were considered to be effective genus-level taxa and given provisional identifiers (e.g., *Leptolyngbya* - Clade I, *Leptolyngbya* - Clade II…). To facilitate the visualization of the OTUs distribution and the composition of the sites, plots were constructed using the packages “ggplot2,” “scales” (Wickham, 2018a,b) and “reshape2” (Wickham, 2017) written in R language (R Core Team, 2018).

### Comparison of the Taxonomic Composition Between Regions

A meta-analysis of a set of 15 libraries of bacterial 16S rRNA gene sequences of biocrusts from North American deserts (Table 2; Velasco Ayuso et al., 2017: NCBI identification numbers PRJNA343817; Fernandes et al., 2018: NCBI identification number PRJNA394792) was used for comparisons. These libraries had been constructed using primers (515F and 806R primers - Caporaso et al., 2012), whereas those in this work used CYA 359 and 781a/b (Nübel et al., 1997). Because of this, the quality-controlled and paired sequence files were merged into a single FASTA file and imported together into the QIIME 22018.2 for analyses. Sequences were clustered at 97% similarity using closed reference OTU picking with VSEARCH (Rognes et al., 2016) with the Green Genes 13_8 database (DeSantis et al., 2006) providing the reference sequences. The resulting OTU table was filtered to only include cyanobacterial sequences. OTUs were then aligned using Mafft (Katoh and Standley, 2013) and a phylogenetic tree was generated using FastTree (Price et al., 2010). Community differences were assessed *via* permutational multivariate analysis of variance (PERMANOVA) performed on Bray-Curtis distance matrices of relative abundance derived from sequencing and used 9,999 permutations. PERMANOVAs were performed using the function “adonis2” in the <vegan> package (Dixon, 2003) run in “R” (R Core Team, 2018). The <vegan> function “betadispar” was used to test the variances (PERMDISP). A *p* of 0.05 was set as the significant threshold for all multivariate statistical analyses. Community composition was visualized with NMDS, using 25 restarts and 9,999 iterations.

**Table 2:** Samples and sampling localities of biological crusts in United States of America deserts used for comparison.

Sample code	Origin	GPS coordinates
11, 12	Sevilleta Long Term Ecological Research, Chihuahuan desert	34°20′N, 106°41′W
14, 17, 20, 24, 28, 30, 31, 34, 36, 40	Sevilleta Long Term Ecological Research, Chihuahuan desert	34°33′N, 106°72′W
Jornada	Jornada Long Term Ecological Research, Chihuahuan desert	32°54′N, 106°72′W
Fort Bliss	Jornada Long Term Ecological Research, Chihuahuan desert	32°43′N, 105°98′W
Burr	Hill Air Force Base, Great Basin Desert	41°10′N, 113°00′W

### Identification of Significant Environmental Parameters

Climatic data were obtained from the public database available at Center for Weather Forecasting and Climate Research (INPE/MCT; http://bancodedados.cptec.inpe.br/). Average annual precipitation (PRE), average annual high temperature (HT), average annual air humidity (AH), and altitude (ALT) were retrieved. Soil temperature (ST) and pH, which were measured in the field, completed the environmental dataset (Table 1). The packages “vegan” (Oksanen et al., 2016), “ggplot2” (Wickham, 2018b), and “gridBase” (Murrell, 2014), written in R language (R Core Team, 2016) were used to relate environmental data to the distribution and abundance of OTUs. After normalizing the OTU table, a redundancy analysis (RDA) was run as a constrained ordination method to search for possible spatial patterns in the cyanobacterial database.

## Results

### Cyanobacterial Diversity From *cerrado* Biocrusts

Altogether, we detected 14,465 cyanobacterial OTUs, of which the 600 most frequently represented comprised 70% of total reads. Rarefaction curves showed that most samples (except CA1, ZA3, and ZB1) reached a plateau, and therefore most of diversity was accessed (Supplementary Figure S2). The samples that did not reach the plateau were still included in the subsequent analyses because they were similar in community composition with other samples from the same localities that did.

**Figure 1:**
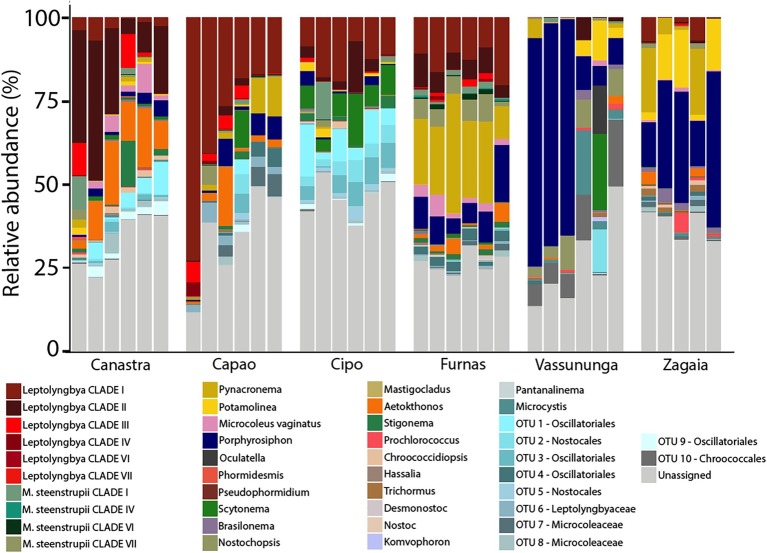
Relative abundance by proportion of 16S rRNA gene sequence reads showing assignable and unassignable to the genus-equivalent level in *cerrado* biocrusts, grouped by sampling locality.

With our taxonomic identification constrained at the genus level, a large portion of the cyanobacterial diversity remained unassigned (*Canastra* 34.5%, *Capão* 19.8%, *Cipó* 46.5%, *Furnas* 9.4%, *Vassununga* 26.9%, and *Zagaia* 35.9%). But a majority of the unassigned OTUs also failed to align to sequences found within the NCBI database. Even among publicly available environmental sequences, such OTUs did not align with greater than 95% sequence identity, speaking for the presence of a significant level of biodiversity novelty in our biocrusts. At this level of resolution, community composition was relatively homogeneous among samples from the same site (Figure 1). OTUs assignable to various clades of *Leptolyngbya* had the most reads. They were dominant in *Furnas*, *Cipó*, *Capão*, and *Canastra*. In some *Capão* samples, sequences allied to *Pycnacronema* were also abundant. The most compositionally divergent communities within our set were those from *Zagaia* and *Vassununga*, where biocrusts were dominated by sequences assignable to *Porphyrosiphon notarisii* Kützing ex Gomont.

**Figure 2:**
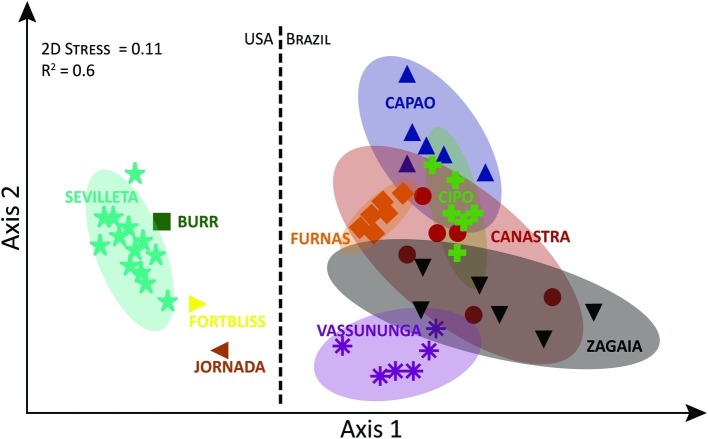
Non-metric Multi-Dimensional Scaling of biocrust cyanobacterial community composition at the OTU level, based on Bray-Curtis similarity including *cerrado* samples and literature-based data for North American biocrusts, for comparison. Ellipses correspond to 95% CI spaces. Data points and ellipses are color coded by locality.

On the basis of OTUs (i.e., independently of taxonomic assignments and including unassigned OTUs), communities also differed significantly in composition (Supplementary Figure S3) among localities but not within them (PERMANOVA, Pseudo-*F* = 5.6904, df = 5/30, *p* < 0.0001), each locality being statistically different from each other in pairwise comparisons (*p* < 0.05). Again here, *Zagaia* and *Vassununga* seemed to be the most divergent *cerrado* localities along a compositional continuum. These differences, however, were much less marked than those found between North American desert communities and those from the *cerrado* as a whole (Figure 2; PERMANOVA, Pseudo-*F* = 16.442, df = 1/50, *p* < 0.0001).

### Identity of Major Cyanobacterial Members in the *cerrado* Biocrusts

**Figure 3:**
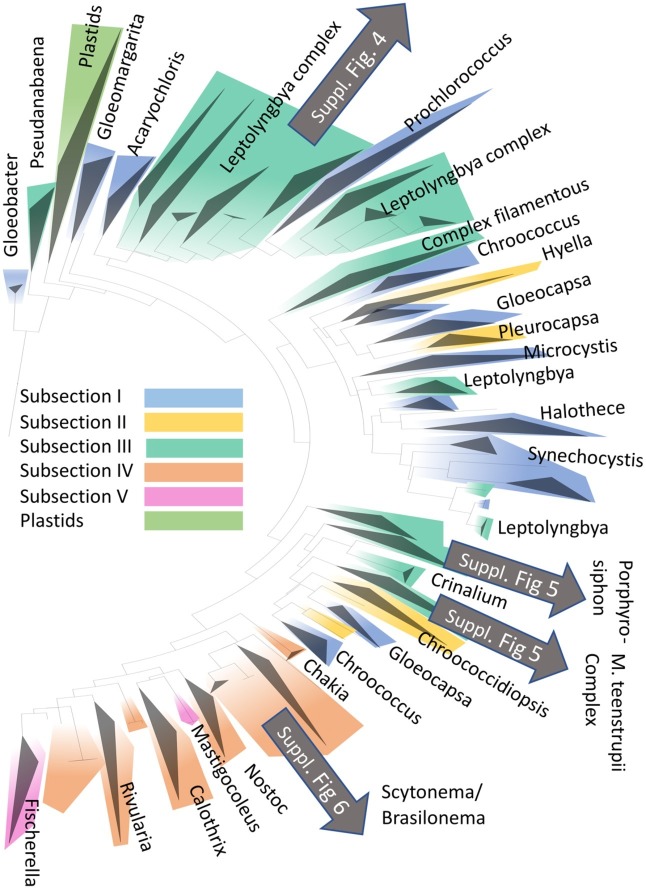
Placement of the more abundant cyanobacteria found in the present study (with greater number of 16S rRNA gene sequence reads; gray arrows) in the phylogenetic radiation of cyanobacteria based on the Cydrasil database. Clades have been colored according to Bergey’s Manual of Determinative Bacteriology’s major morphotypic subsections, and exemplary genera contained within selected clades have been used to label them. Detailed phylogenies of biocrusts OTUs can be found in Supplementary Figures S4–S6, as indicated.

The detailed phylogenetic placement using Cydrasil revealed the occurrence of several important clades of OTUs, which we describe in more detail here. The placement of major *cerrado* biocrusts inhabitants within the cyanobacterial radiation is given in Figure 3. Details for the different taxa are in Supplementary Figures S4–S6.

*Leptolyngbya* Clade I fell within the poorly defined, polyphyletic complex of sequences belonging to thin filamentous cyanobacteria that are usually attributed to this morphogenus and was formed by OTUs related to the public sequence of *Leptolyngbya frigida* (Fritsch) Anagnostidis and Komárek. It was clearly polyphyletic to the clade containing the sequence for the generic type species (*L. boryana* Anagnostidis and Komárek), so it likely represents a new generic unit. A second clade in this complex, *Leptolyngbya* clade II, was composed of OTUs similar to the sequence of *Leptolyngbya* sp. 7FUR (MF109116), but they were also, as *Leptolyngbya* Clade I, polyphyletic to true *Leptolyngbya*. Finally, OTUs placed within *Leptolyngbya* Clade III gathered around the sequence of the type species and are likely *Leptolyngbya sensu stricto*. A tree for the “*Leptolyngbya* complex” with assignments can be found in the Supplementary Figure S4.

A set of abundant OTUs with high representation in some of our localities was affiliated with *Porphyrosiphon notarisii* as judged by close similarity with several new sequences derived from *bona fide* cultures obtained from *Furnas* biocrusts (now permanently added to the Cydrasil database). This clade of sequences is well separated phylogenetically from sequences of other large-celled members of the Phormidiaceae, indicating that they do indeed represent a differentiated generic entity, as the traditional taxonomy would predict (Supplementary Figure S5).

A second complex of biocrust cyanobacteria corresponds to the epithet “*Microcoleus steenstrupii*” which has been recognized as a supra-generic entity in need of re-evaluation, not only because its members are not related to *M. vaginatus* (Vaucher) Gomont ex Gomont (the type species for the genus *Microcoleus*, which falls within the Complex Filamentous clade of Figure 3), but also because it encompasses a variety of separated clades with apparently diverging ecological traits (Fernandes et al., 2018). *Sensu lato*, the complex also includes sequences aligned with cultured strains assigned to *M. paludosus* Gomont, and the recently described *Pycnacronema savannensis* Martins, Machado-de-Lima, and Branco isolated and described from Brazilian savanna biocrusts (Martins et al., 2019). All these cyanobacteria are morphologically indistinguishable from *Phormidium*, except for the fact that they often form trichome bundles. Many OTUs in our crusts clustered within this complex, and given the difficulty in systematics, we have maintained the epithet “*Microcoleus steenstrupii* complex” to refer to them, except for OTUs clearly affiliated with *P. savannensis*, which conformed a major component in some of our crusts (Supplementary Figure S5). Several new full sequences of *P. savannensis* from *bona fide* cultures were included in the database before analyses to ensure that this choice was correct. OTUs in this complex not affiliated with *Pycnacronema* were not very numerous in terms of total reads.

OTUs most similar to the sequences of *Microcoleus vaginatus*, the type species for the genus *Microcoleus* in the Oscillatoriaceae, and likely the most common terrestrial cyanobacterium globally (Garcia-Pichel, 2009), while not very common here, constituted another clear assignment.

Within the heterocytous cyanobacteria, many OTUs were phylogenetically cognate with members of *Brasilonema* sp. (Supplementary Figure S6), previously unreported from soil crusts. Among the three major heterocytous types found in North American arid land crusts (Yeager et al., 2007), only *Scytonema* found significant representation in the *cerrado* crusts.

### Relationship Between Operational Taxonomic Units and Environmental Variables

**Figure 4:**
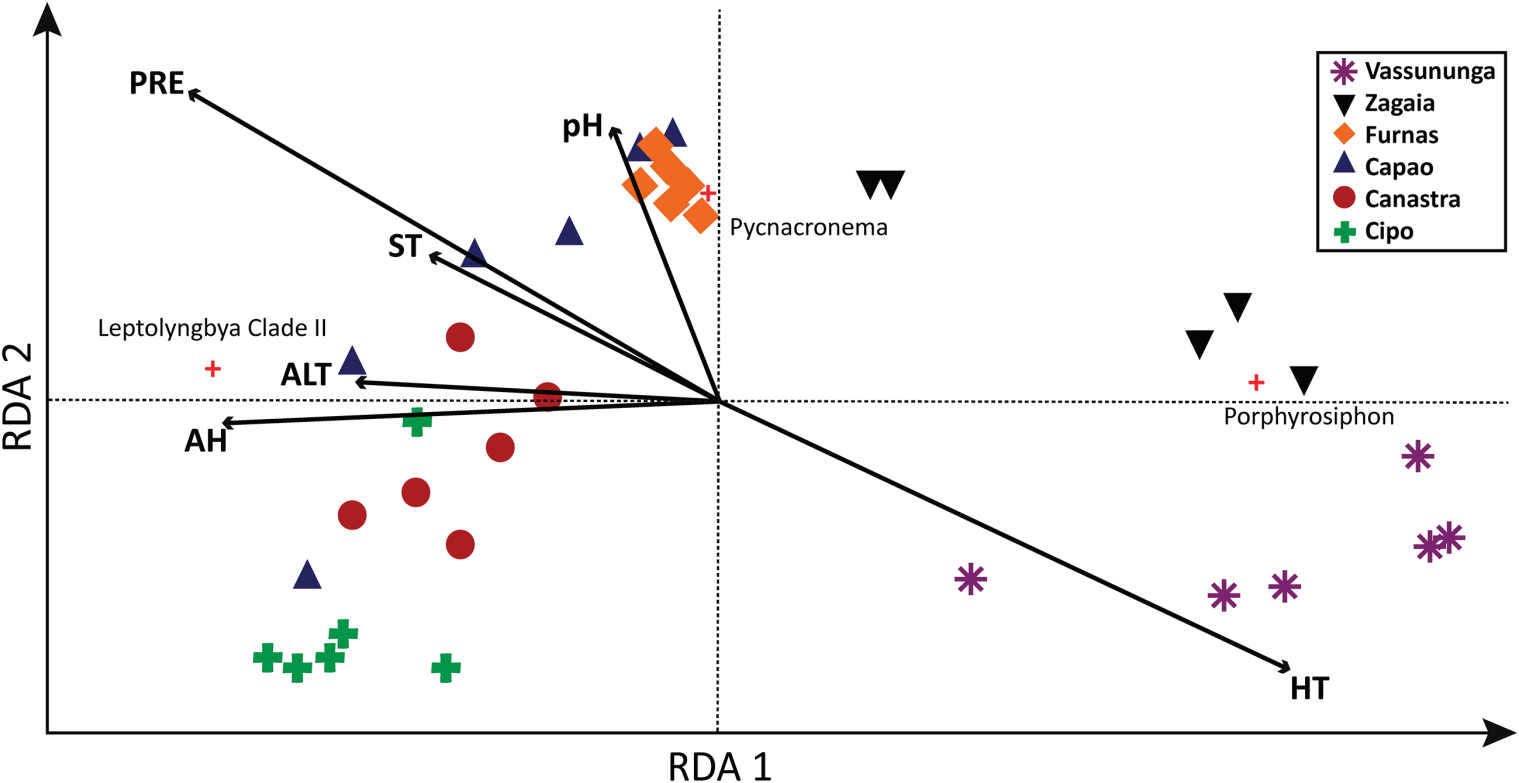
Redundancy analysis (RDA) relating climatic variables with cyanobacterial diversity in 36 sites from six localities in the Brazilian *cerrado*. ALT, altitude; AH, air humidity; PRE, average annual precipitation; ST, pH, soil temperature; HT, average annual maximum temperature.

Our RDA analyses (Figure 4) showed that the combined effect of all environmental variables considered (Supplementary Table S1) could explained 58% of the total variability in cyanobacterial community composition among Brazilian biocrusts. The two statistical axes represented in Figure 4 explained 40% of this variability. The opposing vectors of high annual temperature (HT) and precipitation (PRE)/air humidity (AH) explained the majority of the variation in community composition among locations, particularly separating *Zagaia* and *Vassununga* (related to hotter, drier climate) from the rest. The arid end of this *continuum* was determined significantly by the strong contributions of *Porphyrosiphon*, the opposite end by those of *Leptolyngbya* Clade I. Variability among *Canastra*, *Cipó*, *Capão*, and *Furnas* communities seemed to be driven by soil pH (Figure 4).

## Discussion

*Microcoleus vaginatus*, *M. steenstrupii* Petersen, *M. sociatus* West and West, *Nostoc commune* Vaucher ex Bornet and Flahault, *Calothrix*, *Lyngbya*, *Oscillatoria*, *Phormidium*, *Scytonema*, and *Tolypothrix* are considered the most representative and abundant taxa of cyanobacteria in biological soil crusts throughout the world (Büdel, 2003). *Microcoleus vaginatus* and the “*Microcoleus steenstrupii* complex*”* are dominant in biocrust from North American and other arid lands (Garcia-Pichel et al., 2001; Gundlapally and Garcia-Pichel, 2006; Büdel et al., 2009; Hagemann et al., 2015; Schulz et al., 2015; Dulić et al., 2017). Interestingly, many of those taxa were absent (or present only in low abundance) in the *cerrado* crust. Instead, *Leptolyngbya* and *Porphyrosiphon* were well represented and often dominant in these subtropical biocrusts. Among the heterocytous cyanobacteria, *Nostoc* sp. and *Tolypothrix* sp., typical of the colder climates in arid lands (Yeager et al., 2012; Zhou et al., 2016; Giraldo-Silva et al., 2019, submitted) had no relevance in the *cerrado* crusts, yielding to *Scytonema* and *Brasilonema*. Additionally, many of the cyanobacterial OTUs from this study did not have phylogenetically close representatives within common databases, resulting in a high proportion of unassigned diversity. This indicates that a significant unique component of biodiversity in the *cerrado* remains to be characterized. The notion of compositional idiosyncrasy of the *cerrado* biocrusts is supported strongly by their clear differentiation from arid land biocrusts in nMDS analyses. One cannot principally distinguish from these data if barriers to dispersal or differential selection by environmental conditions are responsible for the pattern. However, the fact that at least some taxa typical of arid land crusts are found at low population density in the *cerrado* would rather support the latter view.

*Leptolyngbya* has been commonly reported in BSCs but rarely appearing as the dominant genus in biodiversity studies (Kaštovská et al., 2005; Alwathnani and Johansen, 2011). It is distributed worldwide (Büdel et al., 2009; Hagemann et al., 2015; Schulz et al., 2015), from glacial areas in Svalbard (Norwegian archipelago – Kaštovská et al., 2005) to desert areas at mid-latitudes (Mojave Desert - Alwathnani and Johansen, 2011). However, it is unclear to which of its many polyphyletic clades each reported occurrence belongs. Likely, the “lumping” morphological approach to this genus conceals significant biological and phylogenetic diversity rather than support the notion that *Leptolyngbya* is a true generalist organism. Based on the results and analyses presented here, separating *Leptolyngbya* sequences into distinct phylogenetic clades may help solve this issue and allow the establishment of an ecologically meaningful systematic treatment.

The recognition of this distinct composition and the high frequency of *Porphyrosiphon* and *Leptolyngbya* clearly speak for the need of investigating these players at the physiological and ecological levels since they will be paramount in establishing a baseline for future understanding of the dynamics of the *cerrado* environment. On the assumption that emergent ecological properties of biocrusts depend on their species composition, it will be necessary to revise locally the paradigms of biocrust function and ecosystem services that have largely been derived from arid land biocrusts. *Porphyrosiphon*, an easily recognizable morphotype because of its bright red sheaths, e.g., has also been reported from biocrusts of the savanna ecosystems of Australia (Williams and Büdel, 2012) and Africa (Ullmann and Büdel, 2003), where it is considered widely dominant over other cyanobacteria. We could relate the presence of this organism in the field with sequences of cultivated isolates, which should enable an easier identification of its global biogeography in the future. *Porphyrosiphon* produces gelatinous sheaths that could bind soil particles playing a similar role to that of bundle-forming *Microcoleus vaginatus* and *M. steenstrupii* in arid land biocrusts (Péli et al., 2011).

OTUs that could not be assigned to genus level but with high number of reads, and for which a sufficiently similar sequence was found to public database entries, were classified at order rank (OTU1 to OTU10). This was preferred (instead of family rank) because familial divisions in *Cyanobacteria* are phylogenetically ill-defined or polyphyletic, and taxonomically unresolved. Most unassigned OTUs, however, represent sequences with low number of reads.

The importance of aridity (including temperature and rainfall) as determinant of variations in species composition within the *cerrado*; however, parallels what has been found in arid land and Mediterranean biocrusts (Garcia-Pichel et al., 2013; Muñoz-Martín et al., 2019). Although it is necessary to consider that the set of variables used in this work was rather restricted, in the present case, *Leptolyngbya Clade-*I abundance was positively correlated with the wetter, least hot sites, while *Porphyrosiphon* showed an opposite trend, explaining their apparent mutual exclusion as dominant forms. *Porphyrosiphon notarisii* was found to be also a dominant species in Australian Mulga Lands (Williams and Büdel, 2012), where it reportedly tolerated and recovered from drought exceptionally well. This success could be, at least in part, related to their copious sheath investments, which are thick and deeply colored with a red extracellular pigment known as gloeocapsin, likely serving a sunscreen role, and which probably decreases the albedo of the soil, increasing its temperature even further (Couradeau et al., 2016).

The biodiversity of cyanobacteria that exists in biocrusts from *cerrado* seems to be distinct from that of other well-known assemblages, such as those from deserts, and in many aspects quite unique. Dominant species in *cerrado* crusts are marginally present, or even absent, in arid lands evidencing the forcing by large-scale climatic patterns. At the same time, biocrusts from relatively close areas within the *cerrado* domain showed variations in composition that are attributed to local, geographically more restricted conditions. The interaction of the conditions on both scales determines the specific characteristics of the communities and may have implications for the ecological services that biocrust may be able to provide to the ecosystem in each locale.

## Data Availability Statement

The datasets generated for this study can be found in the NCBI Bioproject database under the accession number PRJNA381019 (https://www.ncbi.nlm.nih.gov/bioproject/PRJNA381019).

## Author Contributions

NM-L and VF contributed equally to this manuscript. NM-L, JR, and LB designed experiments and sampled the biocrusts. NM-L, JR and VF performed the laboratory research. NM-L, VF, FG-P and LB processed and analyzed the data. DR wrote and executed the codes for bioinformatics analysis. SV wrote the code for the environmental data analysis and executed it. NM-L, VF, FG-P and VF wrote the manuscript. All authors contributed to the discussion of data and finalization of the manuscript. All authors read and approved the final manuscript.

### Conflict of Interest

The authors declare that the research was conducted in the absence of any commercial or financial relationships that could be construed as a potential conflict of interest.
