# Supplementary material for: The Compositionally Distinct Cyanobacterial Biocrusts From Brazilian Savanna and Their Environmental Drivers of Community Diversity
Source: Front Microbiol. 2019 Dec 17;10:2798. doi: 10.3389/fmicb.2019.02798 (PMC6929519; doi:10.3389/fmicb.2019.02798)
Supplement: Supplementary file 1 [file Data_Sheet_1.docx]

Supplementary Material

# Supplementary Tables

Table S1. Measurements of environmental variables for each sample. Altitude (ALT), average annual precipitation (PRE), soil temperature (ST), average annual high temperature (HT), average annual air humidity (AH) and pH.

| **SAMPLE CODE** | **ALT (m)** | **PRE (mm)** | **ST (^o^C)** | **HT (^o^C)** | **AH (%)** | **pH** |
| --- | --- | --- | --- | --- | --- | --- |
| CA1 | 1194.1 | 1616.21 | 18.651 | 29.1 | 78.51 | 6.14 |
| CA2 | 1194.2 | 1616.22 | 18.652 | 29.2 | 78.52 | 5.97 |
| CA3 | 1194.3 | 1616.23 | 18.653 | 29.3 | 78.53 | 6.07 |
| CA4 | 1194.4 | 1616.24 | 18.654 | 29.4 | 78.54 | 6.92 |
| CA5 | 1194.5 | 1616.25 | 18.655 | 29.5 | 78.55 | 6.72 |
| CA6 | 1194.6 | 1616.26 | 18.656 | 29.6 | 78.5 | 5.61 |
| CAP1 | 811.1 | 1572.91 | 35.61 | 28.41 | 76.11 | 6.01 |
| CAP2 | 811.2 | 1572.92 | 35.62 | 28.42 | 76.12 | 5.94 |
| CAP3 | 811.3 | 1572.93 | 35.63 | 28.4 | 76.13 | 6.41 |
| CAP4 | 811.4 | 1572.94 | 35.64 | 28.4 | 76.14 | 5.8 |
| CAP5 | 811.5 | 1572.95 | 35.65 | 28.4 | 76.15 | 6.4 |
| CAP6 | 811.6 | 1572.96 | 35.66 | 28.4 | 76.16 | 6.49 |
| CI1 | 1210.1 | 1572.97 | 26.91 | 28.4 | 76.17 | 6.15 |
| CI2 | 1210.2 | 1572.98 | 26.92 | 28.4 | 76.18 | 6.35 |
| CI3 | 1210.3 | 1572.99 | 26.93 | 28.4 | 76.19 | 6.04 |
| CI4 | 1210.4 | 1573 | 26.94 | 28.4 | 76.2 | 6.28 |
| CI5 | 1210.5 | 1573.01 | 26.95 | 28.4 | 76.21 | 6.08 |
| CI6 | 1210.6 | 1573.02 | 26.96 | 28.4 | 76.22 | 5.89 |
| FU1 | 969.1 | 1718.81 | 26.97 | 26.91 | 69.11 | 6.52 |
| FU2 | 969.2 | 1718.82 | 26.98 | 26.92 | 69.12 | 6.73 |
| FU3 | 969.3 | 1718.83 | 26.99 | 26.93 | 69.13 | 6.49 |
| FU4 | 969.4 | 1718.84 | 27 | 26.94 | 69.14 | 6.39 |
| FU5 | 969.5 | 1718.85 | 27.01 | 26.95 | 69.15 | 6.49 |
| FU6 | 969.6 | 1718.86 | 27.02 | 26.96 | 69.16 | 6.3 |
| VA1 | 626.1 | 1411.91 | 23.31 | 30.1 | 68.11 | 6.14 |
| VA2 | 626.2 | 1411.92 | 23.32 | 30.2 | 68.12 | 6.49 |
| VA3 | 626.3 | 1411.93 | 23.33 | 30.3 | 68.13 | 6.19 |
| VA4 | 626.4 | 1411.94 | 23.34 | 30.4 | 68.14 | 5.0 |
| VA5 | 626.5 | 1411.95 | 23.35 | 30.5 | 68.15 | 5.62 |
| VA6 | 626.6 | 1411.96 | 23.36 | 30.6 | 68.16 | 5.67 |
| ZA1 | 1375.1 | 1616.21 | 22.81 | 29.1 | 78.51 | 6.5 |
| ZA2 | 1375.2 | 1616.22 | 22.82 | 29.2 | 78.52 | 6.57 |
| ZA3 | 1375.3 | 1616.23 | 22.83 | 29.3 | 78.53 | 6.15 |
| ZA4 | 1375.4 | 1616.24 | 22.84 | 29.4 | 78.54 | 5.89 |
| ZA6 | 1375.5 | 1616.25 | 22.85 | 29.5 | 78.55 | 6.0 |

# Supplementary Figures


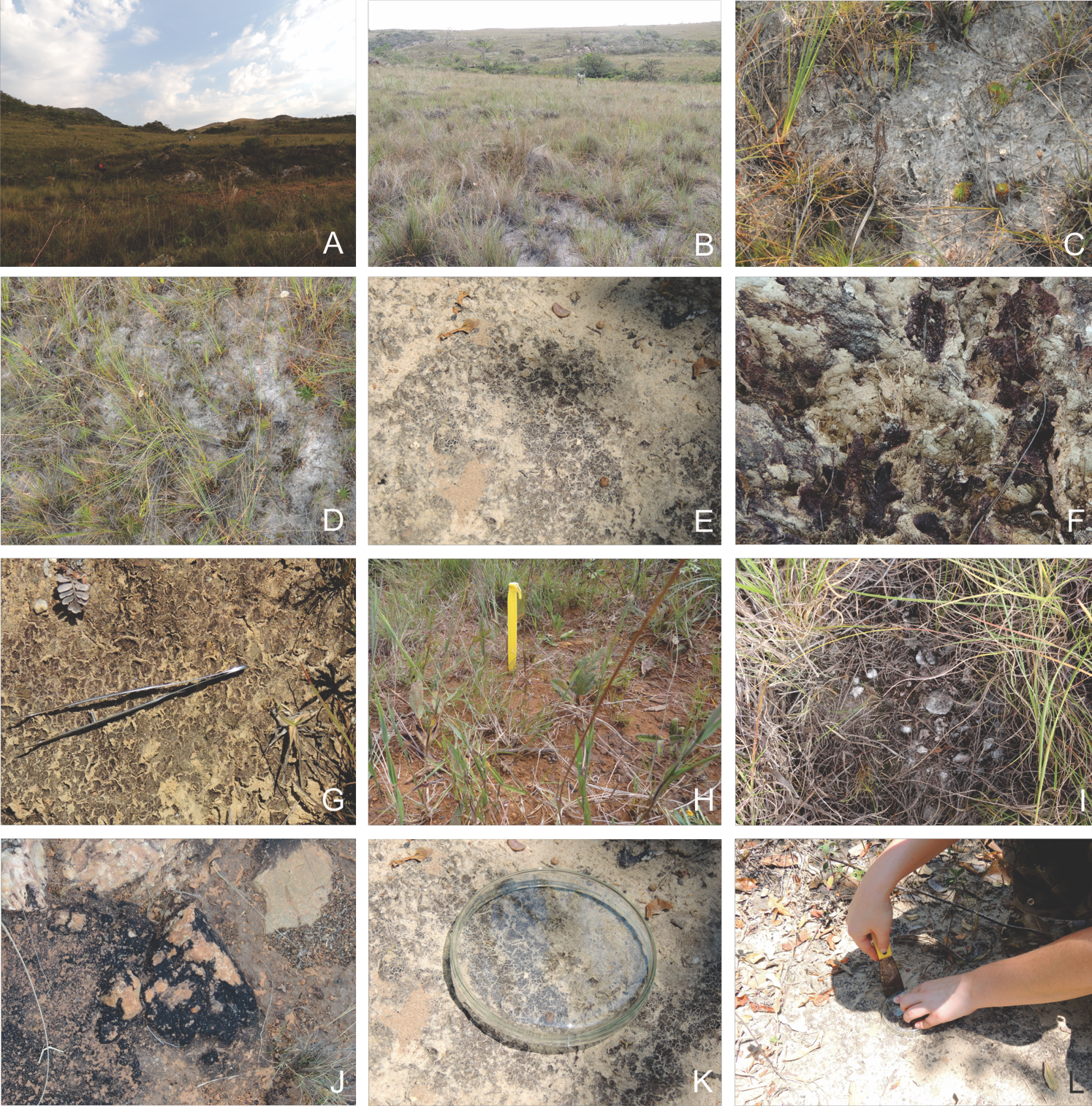


**Figure S1.** Figs. A-L: *cerrado* sampling areas; Fig. A: *Serra do Cipó* National Park (*Cipó*); Fig. B: *Serra da Canastra* National Park (*Canastra*); Figs. C-J: aspect of different biocrusts; Figs. K-L: crust collection method.


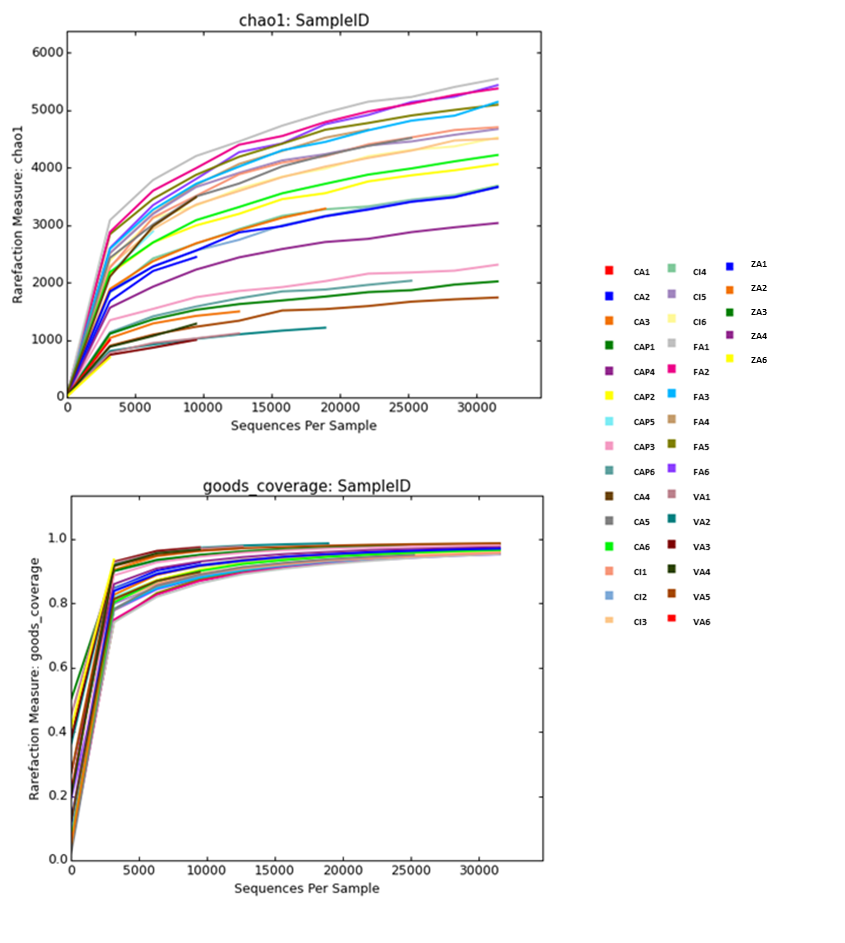


**Figure S2.** Rarefaction curves using “Chao1” (above) and “goods coverage” (below) for the 35 samples from six localities. Codes of sites according to Table 1.


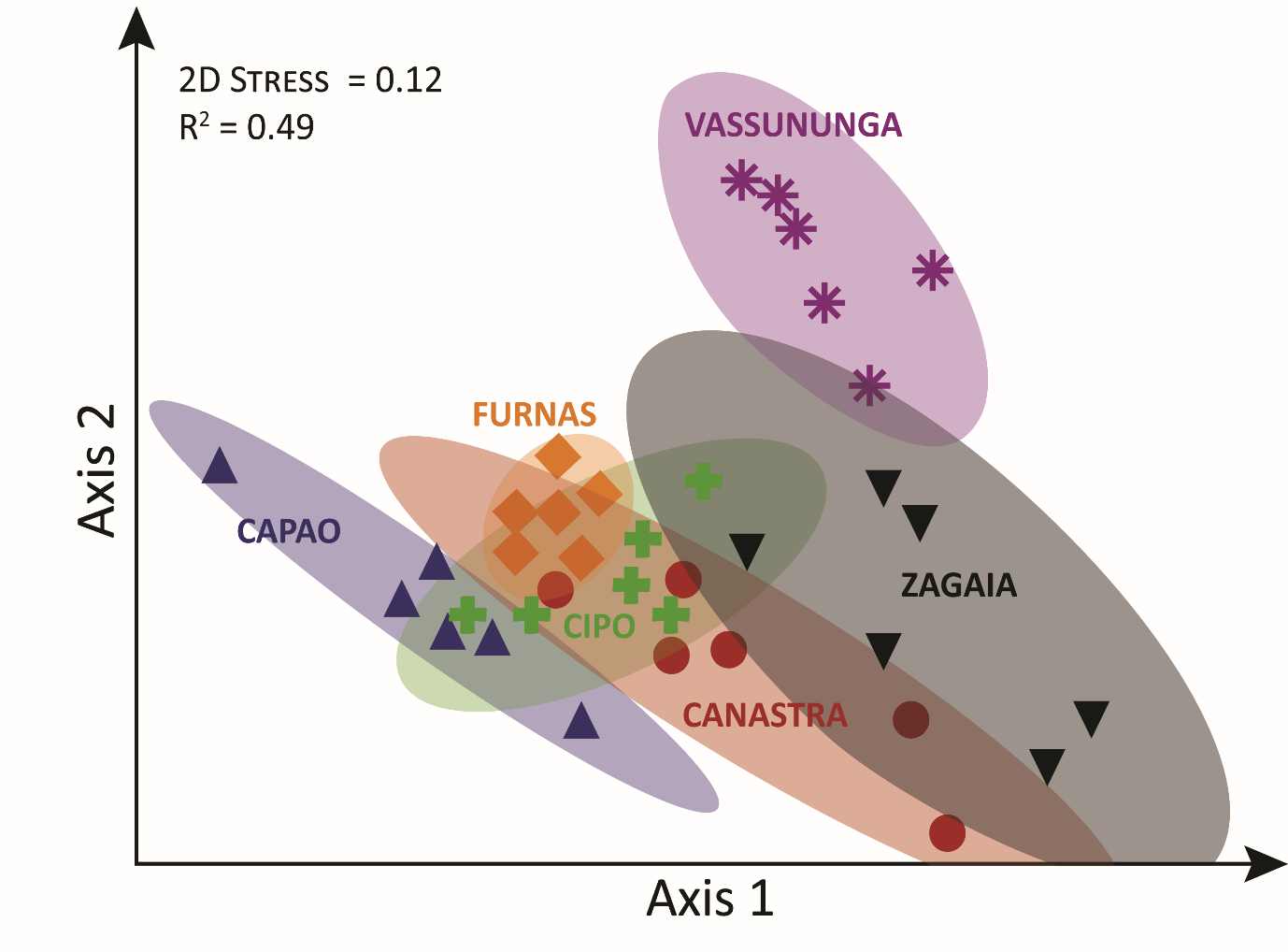


**Figure S3.** Non-metric Multi-Dimensional Scaling of biocrust cyanobacterial community composition at the OTU level, based on Bray-Curtis similarity of Brazilian *cerrado* biocrusts. Ellipses correspond to 95% CI spaces. Data points and ellipses are color coded by locality.

**
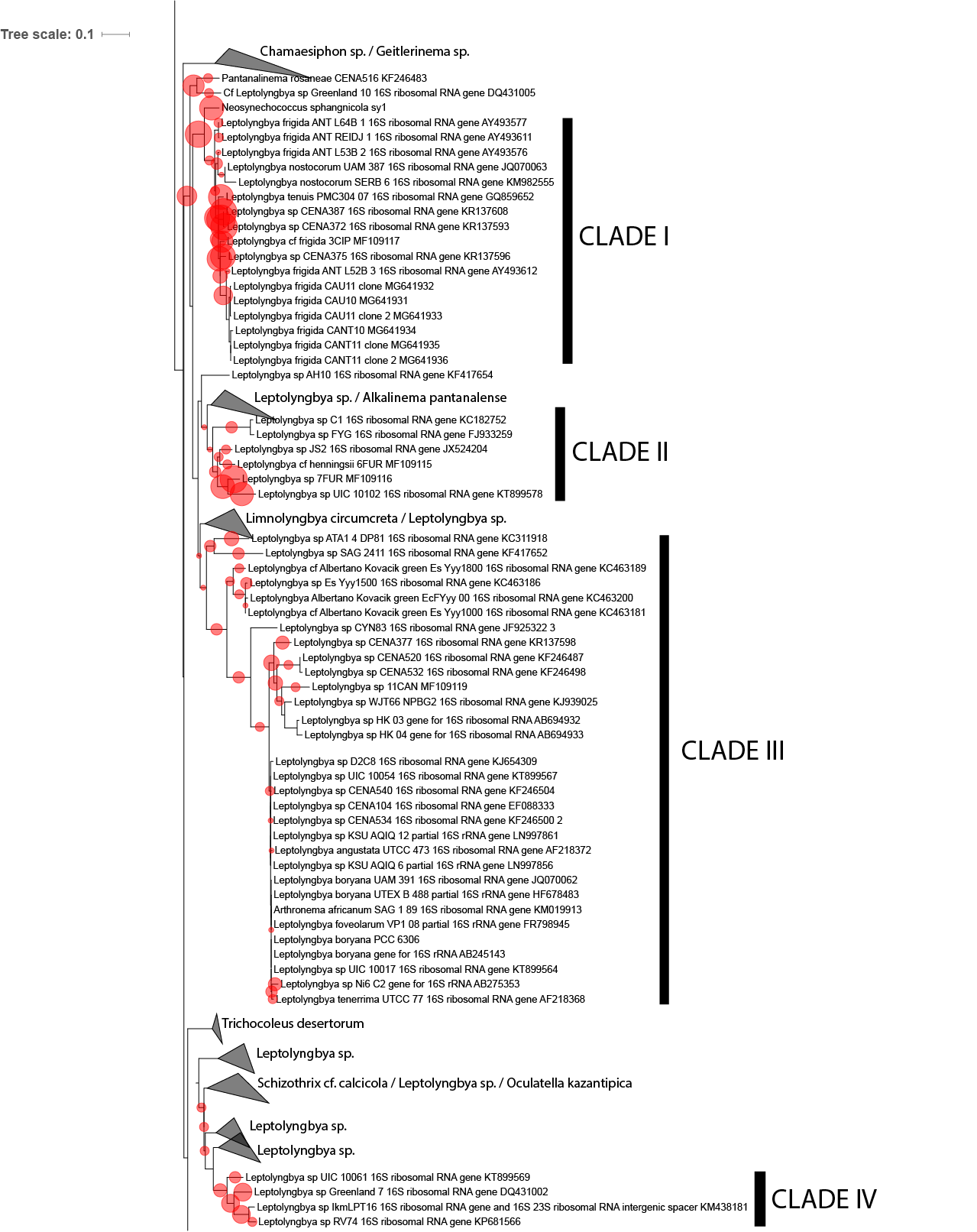
**

**Figure S4.** Phylogenetic placement of biocrust OTU in *the Leptolyngbya* Complex. Red circles indicate most likely placement by similarity, and the size of the circle is proportional to the number of OTUs at the node.


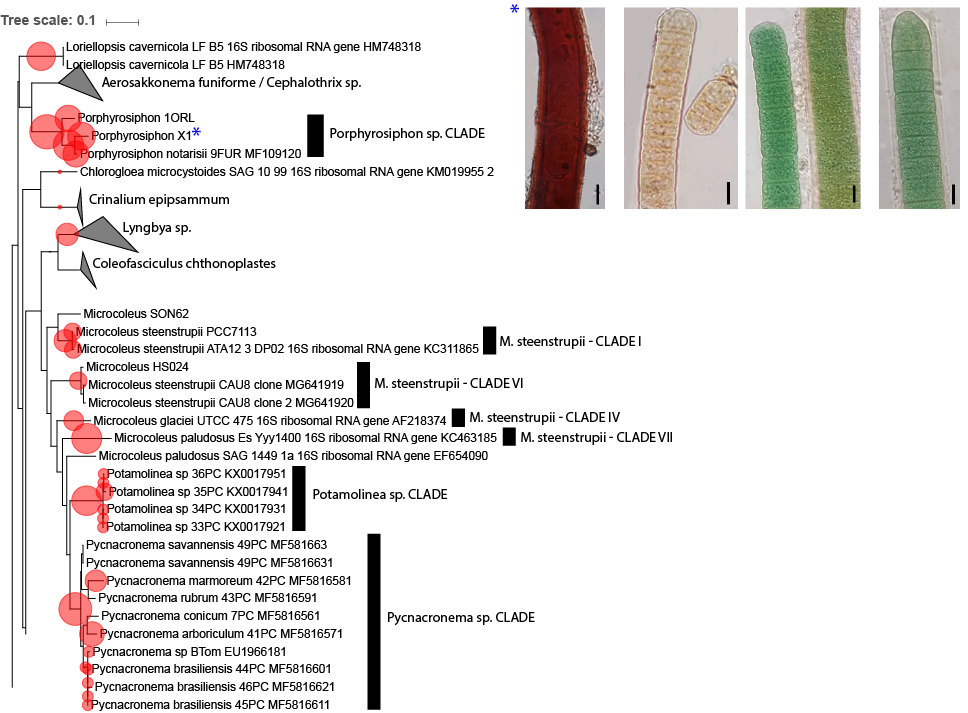


**Figure S5.** Phylogenetic placement of biocrust OTUs in *the Porphyrosiphon* clade and the *Microcoleus steenstrupii* Complex*.* Red circles indicate most likely placement by similarity, and the size of the circle is proportional to the number of OTUs at the node. *M. steenstrupii* clades have been named after Fernandes et al (2018), The database was enriched with sequences from cultivates isolates of *Porphyrosiphon*, *Potamolinea,* and *Pycnacronema*. Photomicrographs of some of these cultures of *Porphyrosiphon* are in the inset (bar indicates 10μm).


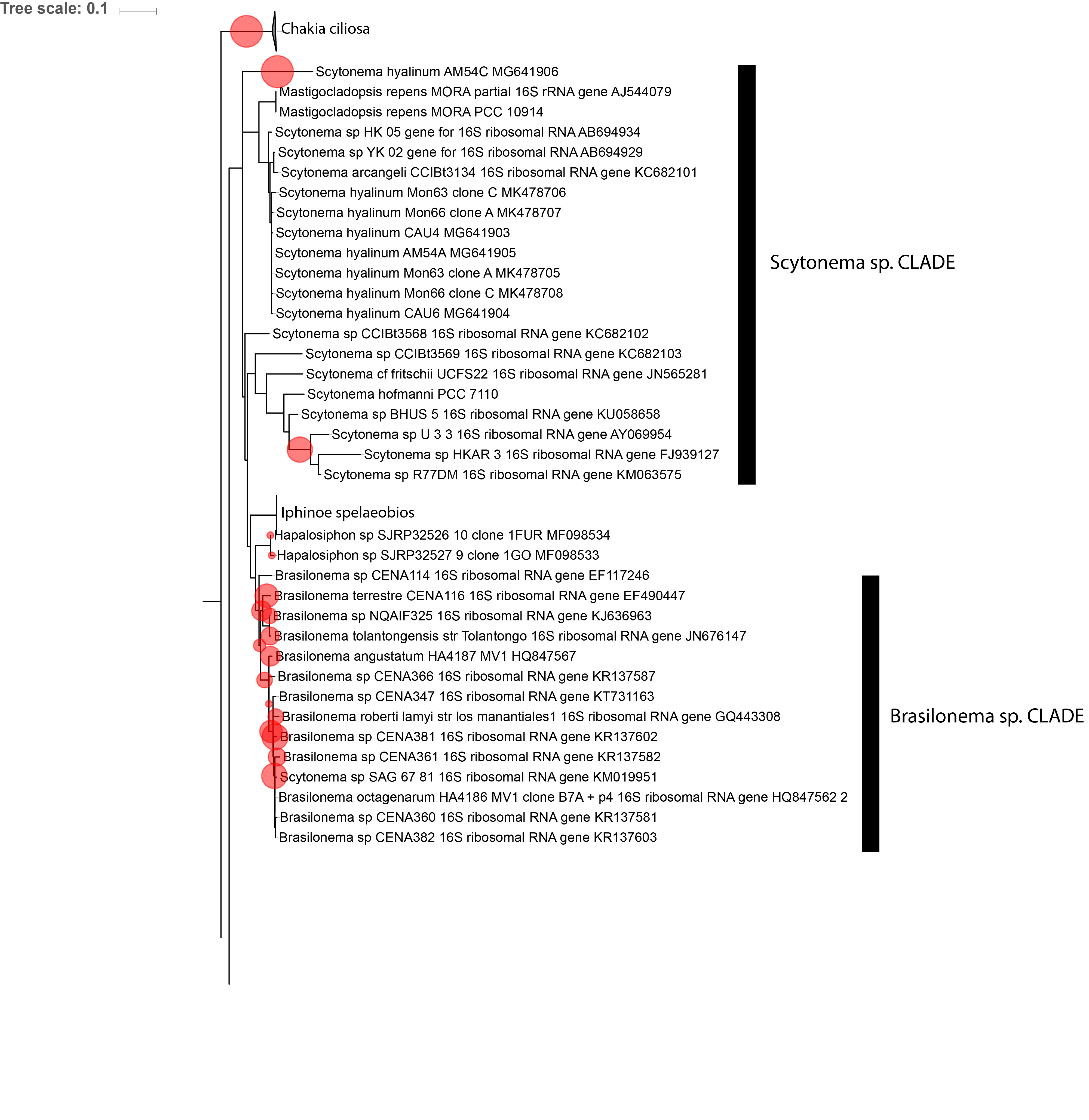


**Figure S6.** Phylogenetic placement of biocrust OTU in the heterocystous cyanobacterial clade including *Scytonema, Brasilonema* and *Hapalosiphon*. Red circles indicate most likely placement by similarity, and the size of the circle is proportional to the number of OTUs at the node.
